# Low-Cost and Data Anonymised City Traffic Flow Data Collection to Support Intelligent Traffic System

**DOI:** 10.3390/s19020347

**Published:** 2019-01-16

**Authors:** Jonathon Handscombe, Hong Qing Yu

**Affiliations:** School of Computer Science, University of Bedfordshire, Luton LU1 3JU, UK; Jonathon.Handscombe@study.beds.ac.uk

**Keywords:** WSN, MAC, slotted ALOHA, beacon, Arduino, Raspberry Pi, GPS (Global Positioning System)

## Abstract

There are many methods of collecting traffic flow data, especially using smart phone apps. However, few current solutions balance the need for collecting full route data whilst respecting privacy and remaining low-cost. This project looks into the creation of a wireless sensor network (WSN) that can balance these requirements in an attempt to negate some of the concerns that come with this type of technology. Our proposed system only collects location data within a defined city area. This data is collected with a randomized identifier, which limits repeated identification of the source vehicle and its occupants. Data collected is shared between vehicle and roadside base stations when the two are in range. To deal with the fluid nature of this scenario, a purposely designed Media Access Control (MAC) protocol was designed and implemented using the beacon-slotted ALOHA (Advocates of Linux Open-source Hawaii Association) mechanism.

## 1. Introduction

Information and data are important to a city’s council in understanding how best to use their available assets and resources. The following research identifies previously explored applications focused on Intelligent Traffic Systems that go beyond merely road usage measurements. They each rely on data generated by systems or solutions similar to that which will be generated by this project’s artefact. The term Intelligent Traffic System (ITS) describes the idea of integrating communications technology with transport infrastructure and vehicles. This integration allows for better management of the available transport systems meaning that they operate more effectively and efficiently [1].

An example of this improvement in efficiency could be the introduction of adaptive traffic light systems. Presently, the timing of many traffic light-controlled junctions depends simply on the time of day. For instance, during morning peak time traffic travelling in one direction is given more priority over those travelling in the other directions. Later, in the evening peak, the timings may be reversed to allow the returning traffic greater priority. During the day and the night, the timings may be fairer, with no priority given to a single direction [2]. With an ITS-based system, the timings could be more dynamic and reflect the traffic that is waiting. The timings can be increased when there is more traffic and decreased when there is less. Which would increase the number of vehicles that are processed by the traffic light system and avoid turning on a green light needlessly if no vehicles are present [3] in the direction. This concept though could be applied to multiple connected traffic light-controlled junctions so that they work in conjunction with each other. Reference [4] proposes a method in which each intersection is in communication with its neighbour. This implementation allows for synchronisation and the creation of green waves, as the authors refer to them as. Successive green lights would be able to decongest busy routes but, as the authors warn, it comes at the cost of greater congestion on other roads. Overall, this demonstrates groups of vehicles could be dealt with more intelligently on a city-wide basis rather than at a single junction.

A further idea that can be applied is that of Priority. Traffic waiting at traffic light controlled junctions is treated fairly, where everyone waits their turn. Not all traffic is equal, though: emergency vehicles, for instance, need to move at speed to deal with an emergency. As [5] notes with queuing traffic comes the difficulty for emergency vehicles that must create a path to progress, slowing them down. The authors present a system in which traffic lights acknowledge emergency vehicles arriving and, as quickly as possible, set the lights in that lane to green. With the traffic flowing, there is a reduced chance that the emergency vehicle will be held up.

A second example of improvement in efficiency is the possibility of congestion detection and alleviation. This mainly applies to long stretches of road, such as motorways, but the idea is to alert road users before they reach the congestion ahead. This allows the road user to make an informed decision on whether to continue or proceed [1]. In the United Kingdom, this example has been implemented on the most congested motorways. These systems are referred to as Active Traffic Management (ATM) and sees gantries placed regularly along the road. Each gantry has a single-lane led matrix display which can alert road users of problems ahead, show lane closures or set the lane speed [6]. In the last two cases, drivers must abide by what is displayed. The changing of speed can slow the rate at which the vehicles arrive at the congestion, this can help to alleviate the congestion and, in some cases, for it to disappear. Research has shown that this type of implementation can have an impact not only on congestion but also air pollution [7].

Citizens using public transport can be seen as another important use case that is positive for a city council, as it lowers the number of vehicles on the road, reduces air pollution and creates revenue. With an ITS-based system, the accurate current and historical traffic data can be applied to improve the service and experience of this method of transport. Focusing on busses, arrival times are usually displayed at bus stops to tell users how many minutes until the next bus. The accuracy of this sort of information is helpful to those which utilise these services. Waiting at a bus stop for a bus that arrives 10 min late, is a frustrating experience for the user. The accurate estimation of arrival times and travel times are potential areas that this technology can be used to provide a better service. Knowing current road conditions ahead, the current location of the bus and historical data from previous journeys allows for the estimations to be fine-tuned and their accuracy improved. This was demonstrated in part by [8], in their research they collected the GPS data from 1213 unique trips made on Dublin’s route 46 A. With this dataset they were able to estimate the time of arrival at the final stop of the route, the accuracy though of the predication improved the further the bus travelled. When the bus was further than 10 km from its final stop, the prediction error was very high. This research demonstrates the possibilities but also the limitations. A solution cannot rely on one source of data (the bus) to correctly estimate travel times and arrival times.

One kind of solution can be to rely upon ITS-based systems which fundamentally require a range of dynamic data collection techniques. Our research including traditional pneumatic road tubes system and induction loop system and new technologies, such as video detection, piezoelectric sensors, and smart app applications, demonstrates four major limitations of current data collective solutions: (1) lacking balances of cost and efficiency, (2) privacy control, (3) incentive publicity, and (4) a new MAC algorithm supporting efficient concurrent communications. 

Especially, the privacy issue has always been ignored. However, privacy cannot be treated as before since more restrictive laws are introduced, e.g., GDPR (General Data Protection Regulation) has been applied to the whole of Europe from May 2018, so a new alternative technique solution must be developed. In the paper “Toward Community Sensing”, [9] acknowledge that for a person to surrender data from private sensors there may be a need for an incentive. They indicate that the incentive for this type of data may need to be of the monetary kind. This brings in the consideration of cost versus reward from the city council perspective especially when budgets must be kept to. It is acknowledged, though, that in some cases the reward may be as simple as those seen in the Active Traffic Management applications. In other words, being able to save time and avoid congestion may be enough of a reward to some users. The city council then, in theory, may need to provide some form of incentive to increase what would be an undeniably slow adoption rate of a system such as the artefact. One such idea may be to offer free or discounted parking charges to those who allow the system to be installed. In Milton Keynes, where our research is based, a smart city in the United Kingdom implemented a type of incentive discount for those who partake in their car sharing scheme [10].

Our initial objective of the project is to carry out systematic research into current data collection limitations. Namely, understanding why information is important to cities and users value their privacy, identify the technical challenges of implementing an alternative solution, and evaluation. As a result, we proposed a WSN-based system and a new Beacon-Slotted Aloha MAC communication protocol as a solution.

The final research contributions of the project are designated a low-cost traffic data collection device that use new Beacon-Slotted Aloha MAC protocol for wireless communications between the tracking device and base station device that supports concurrent and privacy control.

## 2. Traffic Flow Data Collection Methods and Wireless Communication

### 2.1. Traffic Flow Data Collection Methods

#### 2.1.1. Pneumatic Road Tubes

Pneumatic road tubes are simply rubber tubes that are placed across lanes of a road. As the tyres of vehicles run over them the tube is squashed which changes the pressure within. The difference in pressure and the moving air is recorded by a counting device located on one side of the road [11]. This implementation does not gather route data but simply allows for the understanding of how many vehicles use a section of road. 

**Figure 1:**
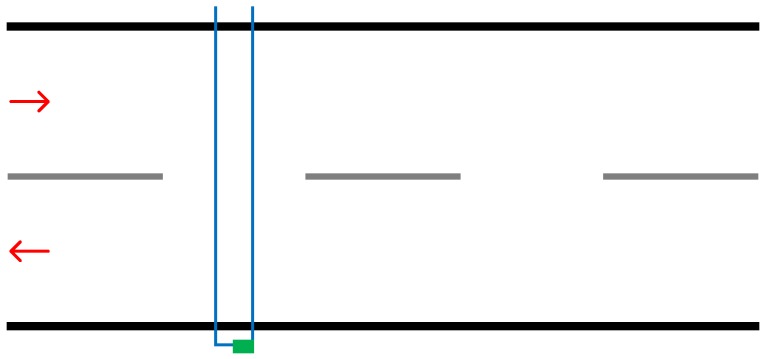
Illustration demonstrating implementation of pneumatic road tubes. The blue lines represent the road tube and the green box representing the counting device.

The implementation of this solution is designed to be temporary and low cost. There are multiple methods by which to lay out the tubes, with each one depending on the road in question [12]. Figure 1 demonstrates a layout where the tube is bolted to each side of the road covering opposing lanes. This implementation means that traffic in each lane will be counted, with no way to differentiate the number of vehicles travelling in each lane. Another layout would involve using a rubber tube and counting device for each lane. In this case, each rubber tube would be terminated on the centre line. 

In addition, some layouts will simply use one rubber tube whilst others will use multiple in succession. Using multiple provides the advantage of being able to understand what direction the traffic is travelling in. If the road does have opposing lanes and is configured in the manner demonstrated by Figure 1, multiple vehicles synchronously running over the tube will be counted as a single vehicle, likewise, vehicles travelling too closely may not be individually counted.

#### 2.1.2. Induction Loop

**Figure 2:**
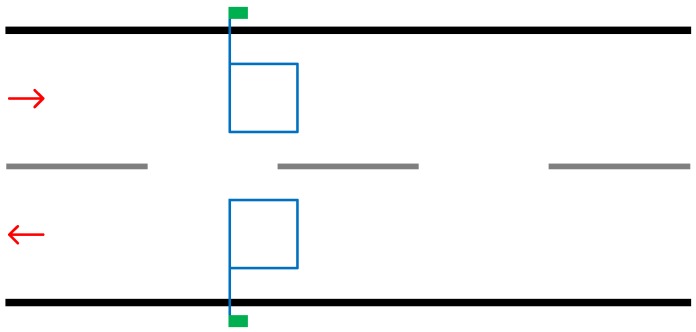
Illustration demonstrating implementation of two induction loops. The blue line represents the wire embedded into the road and the green box representing the power supply and detecting device.

This solution involves embedding wire in a square formation within the road surface. Electrical energy is transferred through the wire at a certain frequency. When a vehicle passes over the loop fluctuations in the frequency occur, these fluctuations are then counted by detecting device located on the side of the road. Some implementations allow for the identification of types of vehicles but, generally, this solution is used only to count vehicles [11]. As Figure 2 demonstrates, the wire is placed within the surface tarmac of each lane which provides a longer-term implementation. The upside of this per lane implementation over the pneumatic tube is that traffic in each lane can be counted individually. This will allow for the tally of vehicles using one lane over another or which direction to be determined.

Placing multiple loops in succession within the same lane allows for the identification of speed and length of the vehicle from which type can be determined [13].

#### 2.1.3. Video Image Detection

This solution uses video cameras to identify the number plates of moving vehicles which are then recorded for later use. This technology is known as ANPR (Automatic Number Plate Recognition) and relies upon optical character recognition. Once a number plate is read, information about the vehicle such as type can be queried from Government databases [14]. From a data point of view, in addition to identifying vehicle type, this solution also provides vehicle counting and the possibility of route identification. The possibility of route identification depends on the number of cameras that exist within a city and their density. It will also depend on the number of cameras a vehicle has passed by to allow a route can be determined or estimated. The implementation of this solution is designed to be long-term, lasting many years. It also comes at a greater cost as a result, as many interconnected video cameras are required.

#### 2.1.4. Piezoelectric Sensors

Piezoelectric sensors turn mechanical energy into electrical energy and are embedded within grooves cut into the road’s surface [11]. As vehicles, bicycles or pedestrians walk over the sensors they generate a small electrical charge which is then recorded by a roadside counting device. The sensors can also be used to measure weight as the electrical charge generated is proportional to the movement they sense.

**Figure 3:**
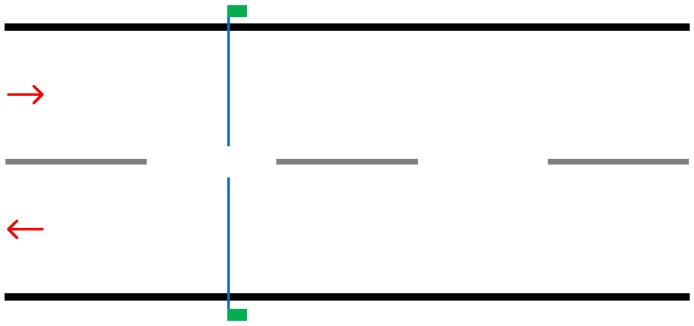
Illustration demonstrating implementation of two piezoelectric sensors. The blue line represents the sensor placed within a groove in the road and the green box representing the counting device.

Like the previous solution, piezoelectric sensors are designed to be a longer-term solution. As Figure 3 on the following page demonstrates, the sensor can cover either the entire width of the road or just a single lane. This presents an advantage over the pneumatic road tube, in that it can collect data from single lanes allowing for analysis of lane utilisation.

#### 2.1.5. Smartphone App

Smartphone app solutions utilise the onboard sensors of a smartphone to gather the required data. This allows for the collection of GPS location data, gyroscope and accelerometer data. The collection of these data types a couple of advantages over the other solutions. Firstly, full location data is collected which will allow the determination of full routes. Secondly, the accelerometer data can be used to help identify potholes or damaged road services [15]. This type of information is useful for the city council, as they can build an understanding of where to spend their road maintenance budgets.

#### 2.1.6. Comparing Results

Table 1 provides a comparison between this project’s system and the other solution explored in the preceding sub-sections. The following data is generalised from the sources provided in those sub-sections.

After researching each of the alternative solutions it is clear that they are each intended for different applications.

In the case of the traditional solutions such as the road tubes, induction loops, and piezoelectric sensors, it is evident that they are limited in their ability. They are more concerned with simply counting or detecting if a vehicle is present and in some layouts: vehicle types. Whilst this will allow the city council to understand how many and what types of vehicles are using a section of road. More data than this is required though for the applications discussed in the literature review. 

The video image detection solution offers more capability as does the smartphone app. The use of Automatic Number Plate Recognition (ANPR) provides an opportunity for the city to determine the routes which vehicles have taken. This does though overlook privacy as no driver will have provided consent for their data to be used in this manner. With the smartphone app, consent may be requested of and given by the user. Of all the solutions discussed the smartphone app offers the greatest functionality and opportunity for the gathering of full route data.

The disadvantage of the smartphone app is the topic of convenience paired with privacy. There are either one or two ways in which the app may be used. Either the user must open the app whenever they make a journey so that data may be collected or allow the app to work always in the background. With the first suggestion, there may be times where the user fails to open the app, so no data is collected. With the second suggestion, the user is asked to give up more of their privacy as anything they do will be collected.

This project will explore respecting privacy by applying a geofence to restrict when data is collected. It will also be restricted further by the fact that it will be constrained to the vehicle. With a smartphone app, even if a geofence is applied to provide restriction data will still be collected when the user is outside of their vehicle. This is the advantage of the project’s artefact system. The user does not have to do anything but drive their vehicle around as they usually would. Once they leave their vehicle they are no longer sharing location data until they return.

Many of the methods are fixed to a segment of a road or to an area. Making modifications to the areas in which they collect data from, would be time-consuming and costly. In the case of the smartphone app and this project’s artefact, the area that is being monitored, the geofenced area, can be modified at will as the area exists in software alone.

### 2.2. Wireless Communication Protocols

Our proposed project is the vehicle tracking node to base station communication, the design of this determines how well the solution will be capable of collecting the data required. 

Most importantly, the solution needs to be designed around the idea that nodes will only be within range for a short period of time. The time may range from tens of seconds to a few minutes but within that period the node must be able to share all its recorded data. It will not be alone though, as there will be other nodes competing to achieve the same goal. This needs to be managed in an effective way to reduce collisions and allow data to be received successfully.

Secondly, the node needs to know that a base station is present. It is not good design to have a node which constantly transmits information whilst knowing that a high percentage of the time it is not within range of anything that can receive it.

Thirdly, the node also needs to know that the base station has received what it sent. Every point of data recorded is important, so care should be taken to ensure that the transmission completes successfully. This is referring to an implementation of acknowledgement and resending.

Finally, the aspect of privacy needs to be taken into consideration after all it is another concern of this project. At no point during this interaction should an individual node and in turn, a vehicle be able to be linked to a previous journey. This then will rely upon the use of unique identifiers that change regular enough that this would not be possible.

To support multiple vehicles and ensure they have an opportunity to share their data, some form of media access control (MAC) protocol was required. 

The decision was made to base the MAC solution on the ALOHA protocol. ALOHA was mainly chosen for its simplicity and adaptability. The protocol can be adapted to meet the requirements needed by the artefact system, namely, a method of both acknowledging and signifying that a base station is near.

To evaluate current possible wireless communication protocols, the 868 MHz variant of HopeRF’s RFM69CW transceiver (HopeRF Electronic, Shenzhen, China) was chosen for the project. The transceiver is low cost, low power and because of the frequency chosen longer range communication is possible. HopeRF states that the transceivers are capable of a 200 m range with no obstacles, which was validated during development. This frequency is, importantly, also unlicensed in the UK for short-range communication [16].

In addition, the transceiver also has the capability to encrypt the data fields of packets and provides cyclic redundancy check (CRC) functionality.

These RF (radio frequency) modules are transceivers meaning that they are capable of transmitting and receiving data but in a half-duplex manner. In other words, they can send or receive but not at the same time. The fact that they can both send and receive is important for this project as both the tracking nodes and base station will need to perform both operations.

The other important factor is their cost, a single transceiver can be bought for around £1.93 [17] from several sellers on AliExpress.

#### 2.2.1. Pure ALOHA

Pure ALOHA is the most basic form of a MAC protocol, it has two rules: 

If there is data to be sent, send the data; and

If, while sending that data, data is received from another node, a collision has occurred. If this happens, try resending the data later.

**Figure 4:**
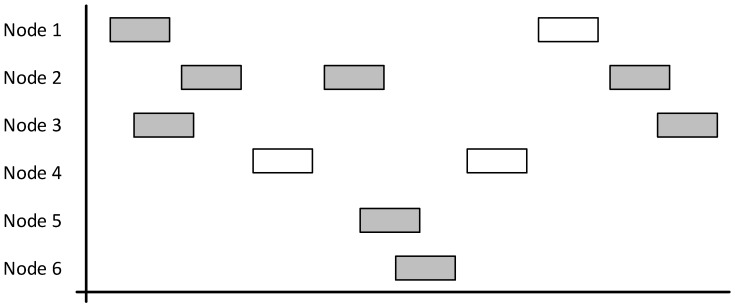
Diagram demonstrating a network using the ALOHA protocol with grey boxes indicating collisions and overlapping. White boxes indicate successful communication.

This type of protocol is intended to be used with nodes that communicate infrequently or when the number of nodes is low. The main problem with this is that the chance of a collision occurring is high, the protocol only has an 18% successful transmission rate, which in turn means that there is increased requirement to resend (as demonstrated by Figure 4). An 18% successful rate is exactly matched to the theory proved by previous research result that the pure ALOHA cannot achieve over and 18.4% successful transmissions rate [18].

Pure ALOHA is not suited to a network such as the one that this project created. Whilst there may be periods of infrequent communication, there will also be bursts of data as vehicles arrive and remain stationary. During these burst periods, the chance of collisions is high. 

Taking the 18% chance of successful communication into account the protocol will only support 17 successful transmissions per second:

(56 kbps/100)×18=10.08 kbps
72 bytes per second = 576 bits per second and 10.08 kbps = 10,080 bits per second
$\frac{10080}{576}=17.5$ messages per second

The problem is that acknowledgements would add to general network traffic. With an 18% chance of successful communication, there is a high chance that the acknowledgements would not make it to the node which requires it. This could force a resend loop, where acknowledgements are never received, and nodes keep resending thinking that the original packet was not received by the base station. With the number of packets that need to send in a short period, this scenario could happen frequently, lowering the throughput.

The other problem with this solution overall is that the vehicle tracking node does not know if a base station exists, which is a requirement for the solution.

#### 2.2.2. Slotted ALOHA

The other important protocol is slotted ALOHA, which modifies the protocol by adding slots that dictate when a node may start transmitting. Adding this rule doubles the throughput of the protocol to a successful transmission rate of 36%.

**Figure 5:**
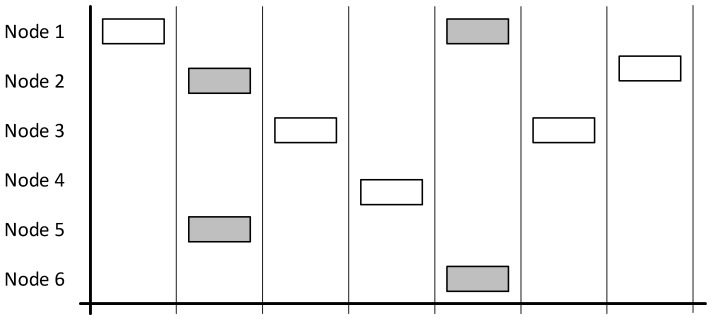
Diagram demonstrating a network using the slotted ALOHA protocol with grey boxes indicating collisions and overlapping. White boxes indicate successful communication.

Figure 5 demonstrates how each node waits for a slot to begin before sending its data. With the increased successful transmission chance of 36%, the network would see 35 successful transmissions per second:

(56 kbps/100)×36=20.16 kbps
72 bytes per second = 576 bits per second and 20.16 kbps = 20,160 bits per second
$\frac{20160}{576}=35$ messages per second

The biggest problem with slotted ALOHA is keeping timings, as the time intervals between the slots are kept by the node. Each node must keep time correctly and ensure that it does not drift enough to start a slot late. With the artefact, keeping accurate time may be difficult. Whilst the base stations can connect to a network time server on the internet to keep themselves accurate, the vehicle tracking nodes will be offline. Keeping accurate timing between all components in the WSN will be difficult.

This solution also presents the same problems as Pure ALOHA, in that it does not provide an opportunity to acknowledge successful transmissions and it also does not signify that a base station exists. If this solution was used, then a slot would be taken up solely by the acknowledgement and could then collide with another packet, causing the same problems as before.

## 3. Proposed Solutions

The WSN created for this project does this by only collecting location data within a defined city area. The recorded data is collected with a randomised identifier, which prevents identification of the source vehicle to provide privacy control and its occupants even through repeated similar journeys. There will be two developed parts to the artefact system which uses low-cost hardware. 

The first part is a GPS tracking node which is fitted to and powered by a vehicle. It will be the device which collects the location data, stores it locally and then shares it with a base station when the two are in range.

The base station, the second part of the artefact system, will collect the information from local vehicle tracking nodes, perform some processing and pass it onwards using an Internet connection to the cloud service. The cloud service would then perform the remaining processing required and store the data. However, this paper focuses on the data collection side.

Finally, the wireless communication between tracking device and base station will be applied to our alternative customized Beacon-Slotted ALOHA MAC protocol to improve the successful data transmission rate. 

### 3.1. The Software Used

To accelerate development on the software side, a library developed by LowPowerLabs [19] was used to provide an interface between the transmitter and the microcontroller. This library provided a flexible programming interface that negated the need to handle SPI (Serial Peripheral Interface) communication which would have required more time.

Whilst various aspects of the library were flexible, there were some limitations, of which were inherited from the transceiver itself.

**Figure 6:**
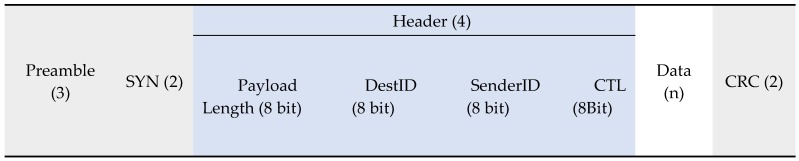
Implemented packet structure.

The packet structure that this implementation uses is shown in Figure 6.

The shading of the fields within the packet is done so to demonstrate the originator. The green fields are handled by the hardware, the blue by the library and the white by this project.

As mentioned previously the transceiver provides an encryption function, if this is used then the payload can be a maximum of 65 bytes. The payload includes the header provided by the library which leaves a maximum of 61 bytes for the data field and 72 bytes overall for the packet.

Each message from a node needs to provide a sessionID, a timestamp, and the recorded latitude and longitude. This amount of data is difficult to fit into 61 bytes and so the following rules were decided upon:

Firstly, the timestamp does not need to be an ISO 8601 formatted timestamp but rather provide only the day, the hour, the minute, and the second. This would provide enough information from which a later process such as one on the cloud service could fill in the unknown. It could add the month and year based on the day in which the data was sent from the base station. There would also be a limited chance of a repeated date within the node’s message queue, for example, the fourth of July and the fourth of August as the node’s data is held only in RAM (Random Access Memory). When the vehicle is turned off any unsent data is lost.

Secondly, the latitude and longitude were represented in decimal form and restricted to five decimal places. This provided a maximum accuracy of 1.1132 m which was the limit of the chosen GPS modules.

These two rules helped to ensure that the required data can be sent within the payload. 

The library also implements nodeIDs which are used to identify senders and receivers for each packet. A Figure 6 explained, the destinationID and senderID fields are each eight bits. Eight bits provides addressing space for 255 nodes, with one of them used for a (base station) and the remainder for the other nodes. This, of course, would not be enough for a production system nor would it be practical as the values are assigned statically. Meaning that if a vehicle is not used for a while, its node will still hold onto an assigned ID, which could have been used by another vehicle’s node.

In addition, a concern of this project is privacy and preventing the identification of individual vehicles. By assigning unique IDs to each node there is an opportunity to identify a specific vehicle. It was due to this concern that the decision was made to use the ID fields for another purpose: to identify vehicles types. With this solution, all base stations are assigned an ID of 1 and a node is assigned an ID based on what type of vehicle it is attached to. For example, busses can be assigned an ID of 2, taxis an ID of 3, and private vehicles 4. There is an opportunity to go into greater detail with this implementation, for instance, 2 represents double-decker buses and 3 represents single-decker buses. In this case, though the privacy concern must be remembered as more detail that is known about a vehicle will make it easier to identify.

### 3.2. The New Beacon-Slotted ALOHA MAC

The solution is then to use slotted ALOHA as a base but introduce beacons which help to signify the beginning of a slot. Each beacon is a broadcast message sent from a base station and provides a timing point for each vehicle tracking node. This will limit the possible time drift and help to synchronise each node.

In addition, the beacons are used as an acknowledgement. Each one contains the sessionID of the last packet it received. This allows the sender to determine the successfulness of its last transmission by comparing the value in the beacon to the one it sent during the last slot.

The final part of the solution is the back-off, which is implemented in a similar fashion to slotted ALOHA. If a sessionID contained in a beacon is not the same as one sent by a vehicle tracking node then it will back-off for a number of slots. When this back-off value has counted down then it will try to resend in the next available slot.

**Figure 7:**
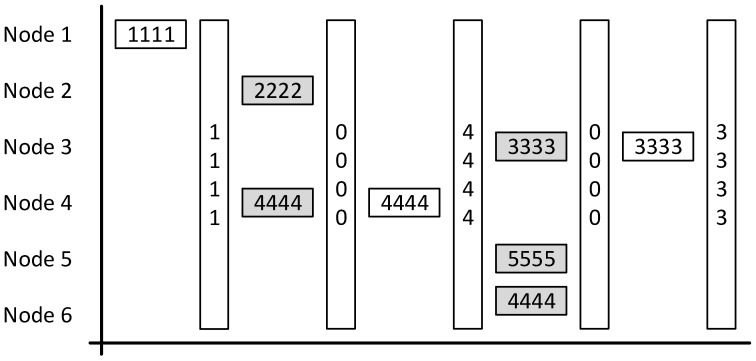
Diagram demonstrating a network using the slotted ALOHA with beacons with grey boxes indicating collisions and overlapping. White boxes indicate successful communication.

Figure 7 demonstrates how the beacon implementation works. In the first slot, only Node 1 sends a packet, which is then acknowledged by the next beacon. 

In the second slot, both Node 2 and Node 4 send a packet each causing a collision. If no packets are received, then a sessionID of 0000 is sent in the next beacon. This tells Node 2 and Node 4 that each of their respective packets failed to arrive at the base station. Node 2 and Node 4 will now back-off, Node 4 picks a 0 slot back-off so tries again this slot whilst Node 2 picks a larger value.

Our evaluation result later shows that the successful transmission rate can achieve 89.6%. 

### 3.3. Privacy Control

The final aspect to discuss is that of privacy and how it will be respected within the system. This statement refers to how the geofence will be implemented and what purpose the sessionsIDs have other than for acknowledgements.

GPS data will only be recorded when a tracking node is within the defined geofence when it is outside then no recording will take place. The aim of this being to limit the data collection to a generalised area in which there is more traffic, lessening the ability to identify individuals. This, of course, depends on where it is placed.

The second aspect of the geofence design is the use of sessionIDs. Each time a tracking node determines that it is within the geofence, it generates a new sessionID which is a value between 1000 and 8000. Whilst it remains within the fence the same sessionID is used, only if the node leaves and then re-enters that a new sessionID generated.

**Figure 8:**
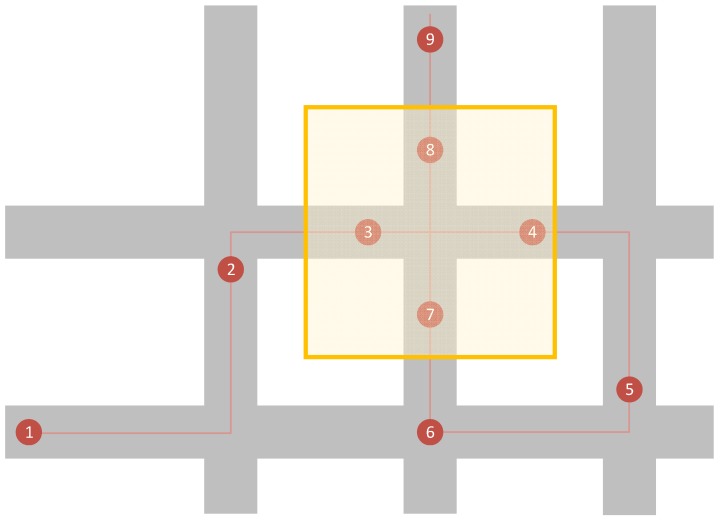
Diagram showing the geofence (yellow area) implementation.

Using Figure 8 as an example, each of the numbered points are GPS collection moments. Whilst there are nine points only the four (3, 4, 7, and 8) within the yellow geofenced area are recorded. Points 3 and 4 will have the same sessionID whilst points 7 and 8 will share a different sessionID. 

This implementation of sessonIDs assists the ability of the cloud service in stitching a full route together as the tracking node may have shared data with multiple base stations. There is the small chance of two tracking nodes picking the same sessionID within a reasonable timespan of each other. It is believed but not proven that further processing could separate the routes in this case based on factors such as entry and exits points into and from the geofence and the type of vehicle using the nodeID within the sent packet.

In the final artefact, the GPS location is checked every five seconds to decide whether it exists within this area or not. This rate of collection and decision ensures that the route taken by the vehicle is correctly identified. However, there does exist opportunities where the GPS signal is interrupted and an incomplete route is recorded. In a situation where two GPS coordinates are many streets apart, a vehicle could have taken a number of different routes between them. For instance, in Figure 8 if point 3 is missing, then there would be difficulty in determining from which direction the vehicle was travelling when point 4 was recorded. Further processing of the data would be required to determine which of the available routes is the likeliest. Ref. [20] presents a method of performing this task by creating a route prediction algorithm that utilises social networking analysis based on historical journey data. Using previous data from the journeys of other vehicles, a reliable prediction could be made and gaps could be filled in.

## 4. Implementation

The focus of the development stage was to turn the designs discussed in the previous section into the real-world system that could be used for evaluation. This chapter documents the two aspects of this stage: hardware and code.

### 4.1. Hardware

The intention of this stage was to verify that the hardware would be able to operate in the manner expected. It was also the intention to create a platform from which the code could be developed upon. 

Later in Section 4.8, we will explain, the initial design was for the artefact system to utilise Atmega328 microcontrollers (Microchip Technology, Chandler, AZ, US). It was for this reason that the following activities were conducted using Arduino Unos which use Atmega328 microcontrollers and ease the development process.

### 4.2. Transceiver

As discussed before, HopeRF’s RFM69CW were chosen for the wireless communication and LowPowerLab’s Arduino library again would be used to accelerate development. 

**Figure 9:**
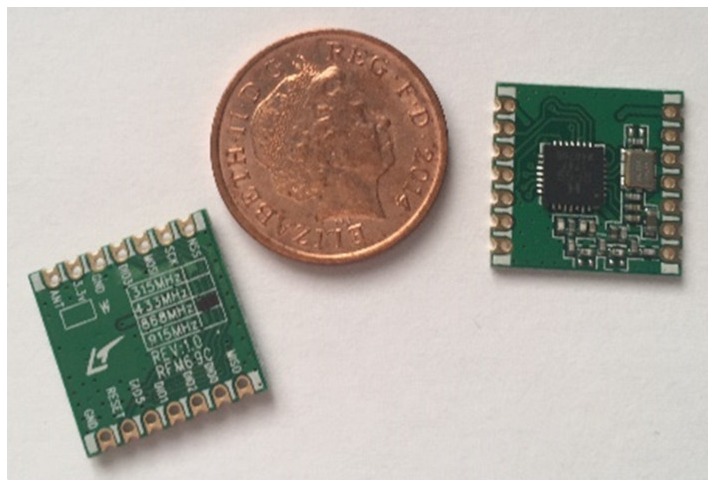
Photo showing two transceivers with a 1p coin.

**Figure 10:**
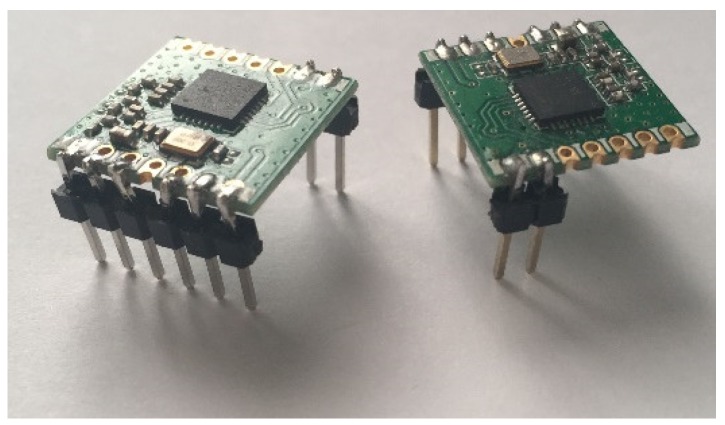
Photo showing a transceiver with soldered on pin headers.

With two transceivers procured the first step was to solder 2.54 mm pin headers on so that the Surface-mount device (SMD) parts could be used within a breadboard. Figure 9 demonstrates the size of the two transceivers and Figure 10 shows the soldered-on 2.54 mm pin headers. Whilst this method of converting the parts from SMD to through-hole components did not produce the cleanest of results, it was successful.

### 4.3. Initial Testing

**Figure 11:**
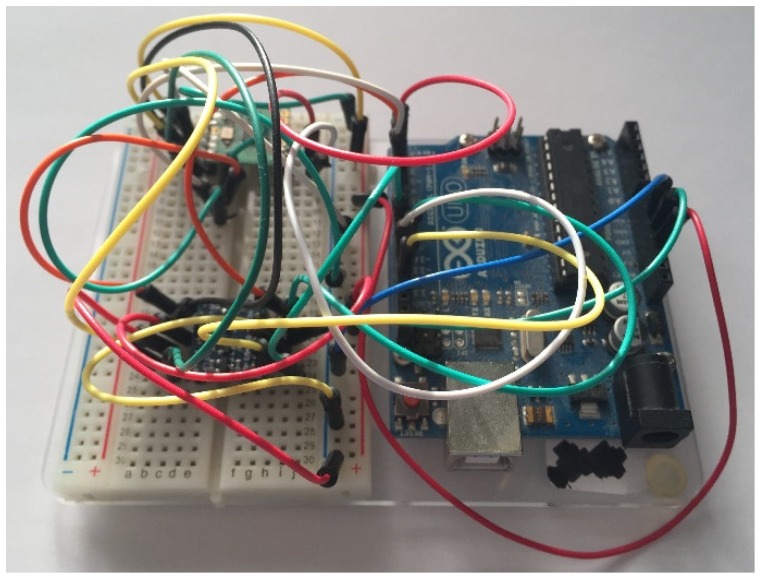
Photo showing the transmitter Arduino Uno.

**Figure 12:**
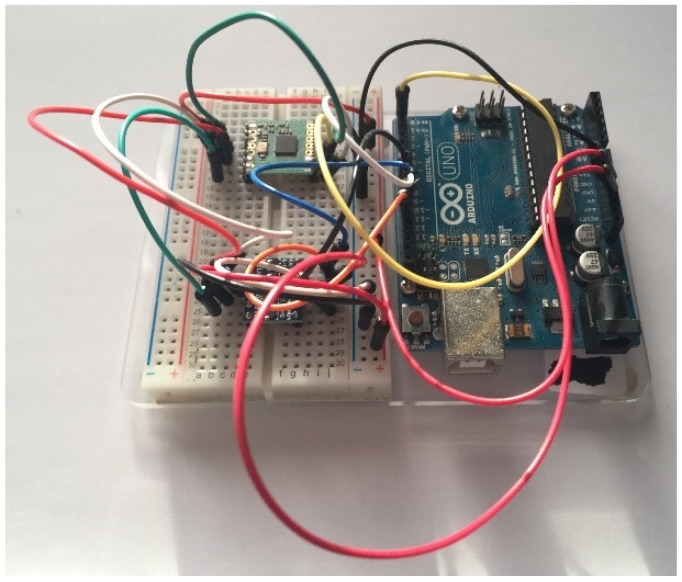
Photo showing the receiver Arduino Uno.

For this initial stage, two separate Arduino Unos (Atmel, San Jose, CA, USA) were used to test both the transceivers and the library. One Arduino Uno played the transmitter role and the other played the receiver role, but both were wired up in the same manner. A logic level converter was used to convert the 5 V Arduino IO pins to the 3.3 V supported by the transceiver. Figure 11 and Figure 12 demonstrate the final configuration.

After uploading the node (transmitter) and gateway (receiver) examples to the respective Arduinos, successful communication could be confirmed from the output displayed in the Serial Monitor. Following this, a short period of experimentation took place to understand the library and what was possible. One notable function that was tested, was the encryption function. By changing the password on one side and not the other, it was confirmed that this functionality worked as expected. Changing the key only on one side made it unreadable on the other.

### 4.4. Range

The final experiment that took place at this stage was concerning the range. Up to this point, the two transceivers had been operating without antennas. This limited their range to around a meter and so an antenna was required to increase this. Following the guide provided by Michael Margolis in the Arduino Cookbook [21], an antenna was created using a stripped solid core wire. The 82-mm long antenna was plugged into the breadboard in the correct position.

This greatly improved the range of the transceiver. Powering the Arduino Uno running the Node (transmitter) example code using a Universal Serial Bus (USB) battery pack a distance of around 30 m was achieved. 

The final range test was to place the Arduino within the glovebox of a car to ensure that the car’s construction was not a hindrance. Watching the output of the serial monitor on the receiver side from 30 m away, it seemed to have no effect.

### 4.5. GPS Module

**Figure 13:**
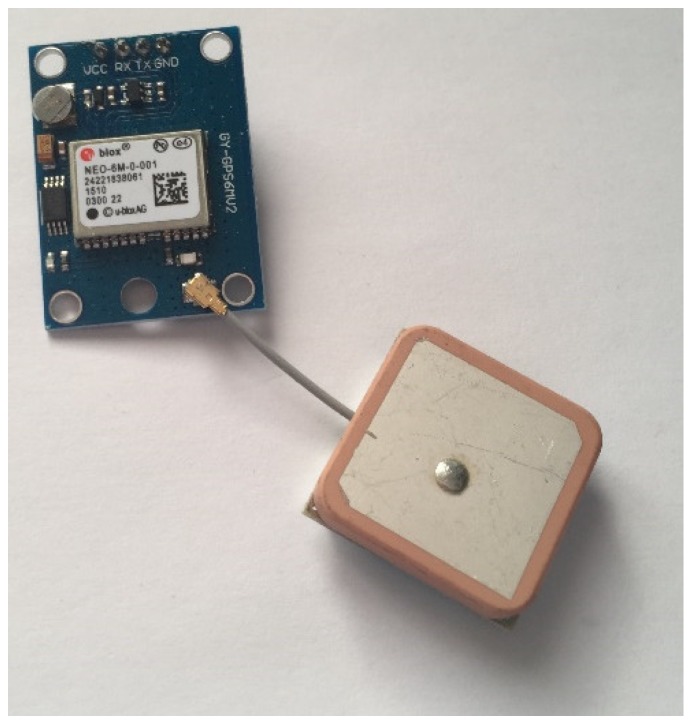
Photo showing the Neo-6M GPS Module with Ceramic Antenna.

Having explored many possible solutions, GPS modules based upon Ublox’s NEO series (Ublox, Reigate, UK) were chosen. The main reason for this choice was their availability in simpler module form and price. For instance, the NEO-6M module purchased again from AliExpress for £3.28, provided a serial input and output. Figure 13 shows the received module.

As the datasheet from Ublox explains [22], the modules use solely GPS owned by the United States government and do not interact with the Russian GLONASS system. The accuracy is up to 2.5 m and the time taken for a cold startup boot is 27 s. The qualities are suitable for this project where cost is of greater concern.

### 4.6. Initial Testing

**Figure 14:**
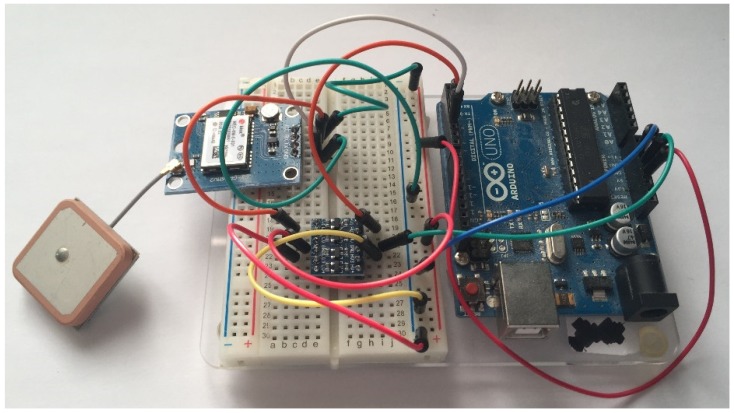
Photo showing the Arduino Uno GPS module testing Arduino Uno.

Using a blank Arduino Uno, the GPS module was connected in the manner shown by Figure 14. 

**Figure 15:**
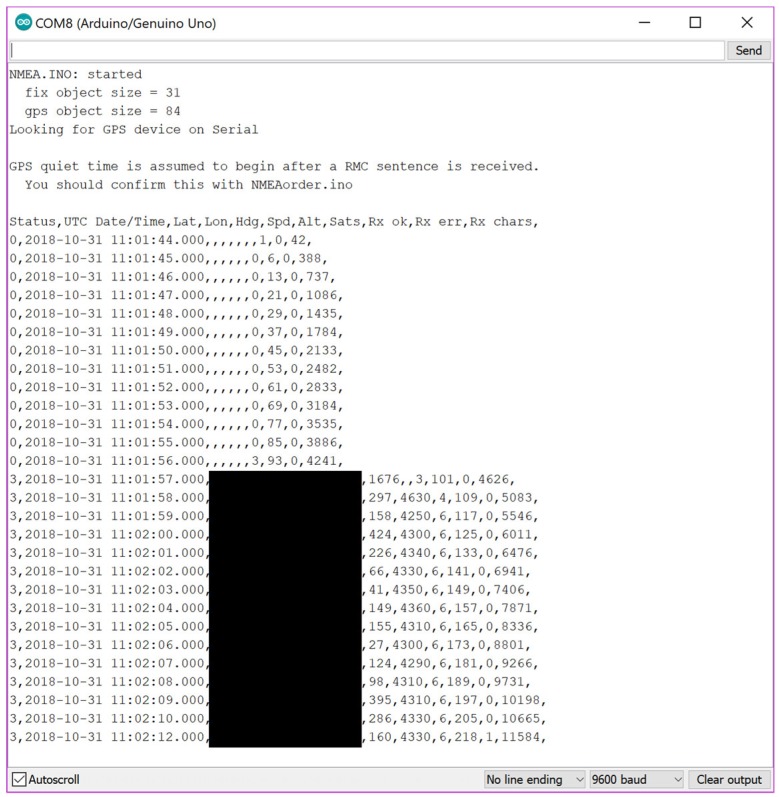
Screenshot of the Serial Monitor showing the output of the NMEA example with redacted GPS coordinates.

The first step was to upload a simple script that outputted what was received over serial from the module to the Serial Monitor. After waiting about 30 s for the module to boot up, messages started to appear in the monitor, which confirmed that the module worked. The module uses the National Marine Electronics Association (NMEA) standard for outputting information and so an Arduino library was sourced to process and clean the output of the messages. NeoGPS was installed and the NMEA example uploaded. Figure 15 shows the output witnessed in the Arduino Serial Monitor.

### 4.7. Interference

So far, the testing had been conducted inside a standard British home which provided confidence in the module’s ability to gain a connection even though obstacles. To confirm this confidence, once again the powered Arduino was placed into the glovebox of a car to ensure that it could lock onto enough satellites to operate with an appropriate accuracy. 

Later testing conducted demonstrated the limitations of the module. Within a building that is made of concrete and steel, the chances of locking onto enough satellites are low. This was not deemed a problem though as, within the real-world context of the system, this would only likely become a problem if the vehicle entered a tunnel or a multi-storey carpark. On normal roads, the GPS module would work as expected, which is demonstrated in the following section.

### 4.8. Together

With the two hardware components successfully tested separately, the next step was to implement the final designs by bringing the components together. This process, as the following paragraphs will explain, presented problems that required changes to the final implementation. The initial implementation for the artefact system was to use microcontrollers on both the vehicle tracking node and the base station.

On the vehicle tracking node, the plan was to use an Atmega328 connected directly to both the GPS and transceiver modules. The microcontroller receives and processes the incoming data from the GPS module and then if valid, stores it in the data memory (RAM). It also handles all communication by identifying and responding to beacons when they are present.

The base station was to utilise both an Atmega328 and a Raspberry Pi. In this configuration, the microcontroller would handle all transceiver module operation including the broadcasting of beacons. The Raspberry Pi would run a python script to handle replies and perform final processing before sending the data to the cloud service. The microcontroller and the Raspberry Pi would be connected using serial communication allowing for bi-directional communication and sharing of data. 

The major problem is that the GPS modules rely heavily on serial communication, sending a single character at a time. This activity occupies the limited data memory (RAM) available to the running program. At the same time, the transceiver library requires data memory to perform its operations. When the transceiver send code was added to the programme it caused the microcontroller to lock up and reset continually. Dealing with these two combined demands of serial and SPI communication was too much for the limited resources of the microcontroller.

### 4.9. Final Implementation

Having had previous experience with the serial communication of the Atmega328, it was identified beforehand that a problem may present itself and so a lesser preferred backup solution was planned. The solution saw the microcontrollers removed from the circuit completely and instead have both sides utilising a Raspberry Pi. Whilst the initial design was overcomplicated, in some ways, the use of microcontrollers would be better for communication as timings could be more precise and reliable. On the other hand, implementing the desired functionality was simpler to do in Python than C. 

**Figure 16:**
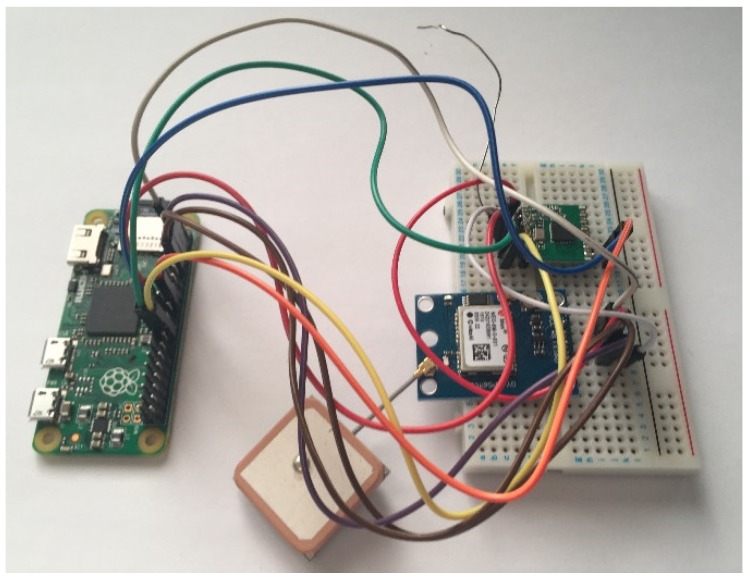
Photo showing the final vehicle tracking node.

**Figure 17:**
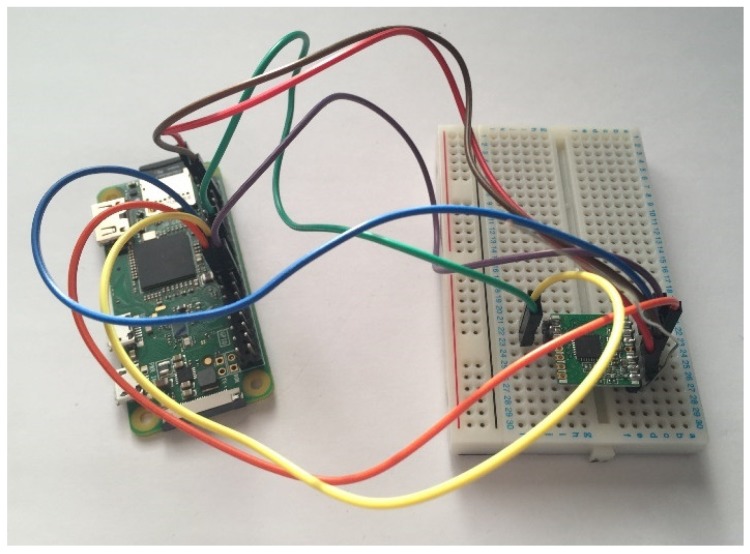
Photo showing the final base station.

Figure 16 and Figure 17 show the final physical form of each of the two artefact components. 

The GPS module on the tracking node is connected to one of the Raspberry Pi’s serial communication pins, allowing for it to operate in the same manner as on the Atmega328. For the transceiver though, a new library was required to create an interface between the SPI module and the Python script that would orchestrate the functionality. A library by GitHub user jkittley [23] was selected as it is a port of the LowPowerLab Arduino library, which meant that it operated in a similar manner and plenty of documentation existed to aid development.

### 4.10. Code

With the hardware finalised, work on the project moved onto developing the Python scripts that would run on the artefact system. Once each of the following Python scripts was developed, a start-up script was written so that the Python code in each instance was executed once the Raspberry Pi’s Raspbian Operating System had booted up. This meant that the Raspberry Pi could run headless without the need for a screen or keyboard, once it was powered.

#### 4.10.1. Vehicle Tracking Node

The code is broken down into four separates but simultaneously running threads:

1. Beacon Listener: The role is to process the content of beacon packets, recording the timestamp contained within and comparing the sessionID against the last packet sent. If sessionID is the same, then the last message has been acknowledged and can be removed from the queue. If not, then Python’s random library generates a back-off value inclusive of zero and five.
2. Queue Handler: The role is to reply to recent beacons if still within the last recorded beacon period, back-off is zero and items exist within the queue.
3. GPS Listener: The role is to handle incoming serial data from the GPS module and pass it to the processing library.
4. GPS Handler: The role is to process the GPS data every five seconds, checking whether the coordinates exist within the geofence. If they do exist, then the coordinates and timestamp based on the time received from the GPS satellites are added to the queue.

Apart from the library used for the transceiver mentioned in Section 4.2, a library is also used to handle the incoming GPS serial data called MicropyGPS [24].

#### 4.10.2. Base Station

The code is broken down into two separates but simultaneously running threads:

1. Beacon Sender: The role is to broadcast a beacon packet every 200 ms. The packet includes a timestamp and the sessionID of the packet received during the last beacon period.
2. Data Receive: The role is to process any data received during the last beacon period. It will update the sessionID to match the last packet received or if nothing was received then a zero will be provided.

## 5. Evaluation

**Figure 18:**
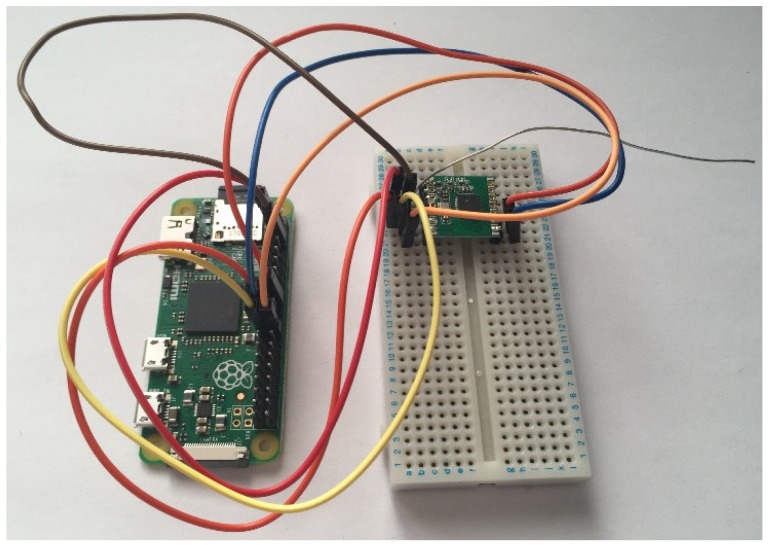
Photo showing the Traffic Generator Node.

To correctly evaluate the system and understand whether the project’s objectives were met, two different experiments were conducted. For each of these experiments, a second vehicle tracker node was constructed, which will be referred to as a traffic generator node. The aim of this node was to run a Python script that would randomly generate network traffic, the constructed node can be seen in Figure 18.

With each beacon, the script either picks a 0 or a 1 randomly using the Python random module. If a 1 is picked, a message is sent or is a 0 is picked the beacon is ignored and nothing happens. Having this secondary node on the network creates a random pattern of traffic, which takes opportunities away from the main vehicle tracking node. This creates an environment similar to that which would be found should the system be used in the real world where other vehicles are present.

### 5.1. Static Experiment

The objective of the static experiment is to understand how efficient the media access control (MAC) protocol is. The efficiency is concluded based on how many slots are utilised and how many resends are required by the vehicle tracking node. This will help to understand how many points of data a vehicle can share based on how long it remains within range.

For the experiment, the vehicle tracking node and the traffic generator node were all placed within a meter of the base station, their distance never changing. 

The vehicle tracking node ran an altered Python script that started with GPS data collection disabled and 50 items in the queue to send. A stopwatch was started when the Python script was executed and stopped when the script had completed sending all 50 items. From each test, the Secure Shell (SSH) terminal output was exported to a text file and data derived. In all, six experiment periods were conducted.

For the first three experiments, just the vehicle tracking node and base station were active with the traffic generator node unpowered. Table 2 shows the results of each three experiments in terms of seconds taken to send all 50 messages.

The time for each of the three experiments was similar and the average was respectable. The system would only require a vehicle that had 50 data points in its queue to sit stationary for around 19 seconds. The MAC protocol is far from efficient, though, with a beacon occurring every two hundred milliseconds and with 50 packets to send, if the MAC protocol was 100% efficient and communication was successful for each beacon period, a perfect time for this scenario would be ten seconds. The average time is almost double this perfect time, which would indicate that beacon periods are being missed and or data is needing to be resent on a regular basis.

For the next three experiments, the traffic generator node was powered and running the network traffic generating Python script. Table 3 shows the results of the final three experiments.

The rate at which the network traffic generating script decides to randomly send can vary massively. With experiments 4 and 5 the times are not that different to those seen in the first three experiments, but with experiment 6 the time triples. During this experiment, the script was sending more packets which were causing the vehicle tracking node to have to back off and try again multiple times. The sixth experiment is reflective of a situation where there are more than two other vehicles waiting at a junction sharing data.

Whilst with the first three experiments the time needed was respectable at twenty seconds, with the sixth experiment, 62 s may be too long to expect a vehicle to wait.

Performing analysis on each of the SSH terminal outputs from all six experiments provides understanding as to what problems occur with the MAC protocol. Table 4 and Table 5 show the data from each perspective whilst the source data can be found in Appendix A.

From the base station perspective, the usage of slots increases in line with the amount of traffic on the network hitting a high of 89.6% efficiency. 

From the vehicle tracking node perspective, the beacons missed is an important number. An unrealised occurrence during the design stage is that missing a beacon also misses an acknowledgement. It was blindly assumed that a node would always see every beacon when it is in range. This has the knock-on effect that the node must go through the back-off period and attempt to resend even if the packet was acknowledged by a base station in a previous beacon. This leads to wasted time and further network traffic.

Remembering that the task is to send 50 packets, with every experiment besides six, the resend total whilst higher than expected is still reasonable based on the real-world expectations. Experiment six, which saw the increased network traffic, sent more than double the number of packets that was originally in its queue (105), which again comes down to missing beacons.

Finally, the back-off method relies on Python’s random module to pick a value inclusive of 0 to 5. Most of the time, the value 0 was picked which the reason for is unknown, but it had an impact.

One of the objectives of the project was to design a method of communication that allowed for opportunistic uploading of data whilst being able to handle multiple vehicles. This static experiment helped to evaluate how efficient the purposely designed MAC protocol was. In doing so it clearly demonstrated that certain aspects of the protocol need improving or even rethinking.

Missing a beacon has a large impact on how much data a node can share. The reason why beacons are not always seen is unknown but the probability of one being missed increases with the amount of traffic on the network.

It is believed that moving the transceiver interaction from a microcontroller to a Raspberry Pi may be a factor. Further research needs to be conducted using a microcontroller capable of handling both the GPS module and the transceiver to determine whether this belief is true.

### 5.2. Dynamic Experiment

The objective of the dynamic experiment was to understand how well the artefact system worked in its intended setting. The experiment was designed to test the geofence implementation and to further test the MAC protocol by having the vehicle tracking nodes move and not remain static. 

**Figure 19:**
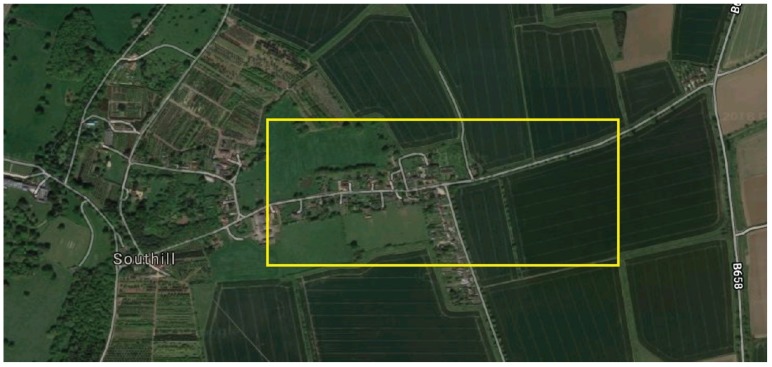
Screenshot of Google Maps showing the geofenced area [25].

For the experiment, a 1 km^2^ geofenced area was defined (Figure 19). The top left coordinate for the geofence is 52.066949, −0.322318 and the bottom right is 52.063082, −0.307944.

Within this area, a quiet T junction, shown in Figure 19, was selected that enabled two scenarios to act out. 

The first scenario entitled ‘Drive By’, sees the vehicle driving past the junction at the 30 miles an hour speed limit. The yellow line in Figure 19 represents the route taken in a right to left direction.

**Figure 20:**
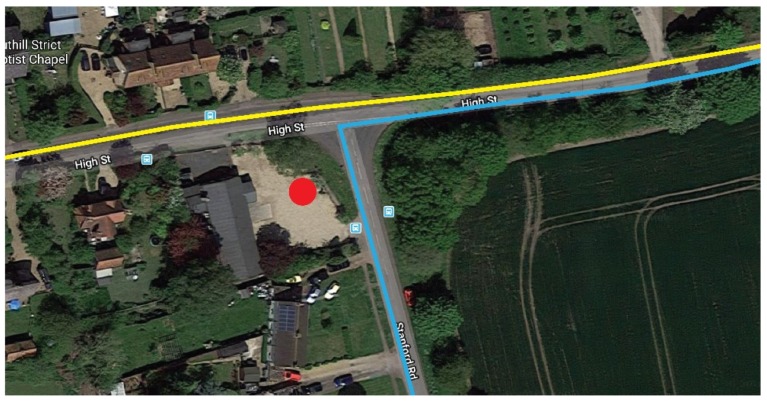
Screenshot of Google Maps showing routes taken and location of the base station [26].

The second scenario entitled ‘Stop’ involves the vehicle coming to a full stop for ten seconds before driving off. The blue line in Figure 20 represents this scenario, with the direction of travel being from the bottom of the image to the right.

With each scenario, the vehicle tracking node was kept inside the glovebox of the moving car. The base station remained static in the location shown by the red dot in Figure 20. 

With both scenarios, the vehicle had to loop round local roads to get into the correct position, which took time and so overall only four loops where performed. This provided an opportunity for four Drive By scenario experiments and four Stop scenario experiments, eight in total. For only the final loop, experiments 7 and 8, the network traffic generating node was used, to again create a more realistic situation.

Finally, between each experiment, the vehicle tracking node’s Python script was restarted to ensure that the queue was empty and the SSH terminal output could be easily saved. The full data from each experiment conducted can found under Appendix B.

#### 5.2.1. Geofence

Looking firstly at how the geofence implementation worked, Table 6 shows how many coordinates were recorded.

Based on the results of the experimentation the geofence implementation works as intended. Every five seconds when the current location was checked and only those that were within the defined geofence were added to the queue. At no point during any experiment was a coordinate added that existed outside of it. In addition, as was intended, a new sessionID was generated every time the vehicle entered.

The difference in the number of recorded coordinates between the two scenarios is the down to how the geofence was applied. If a vehicle was driving past the junction, then it remained for more time inside, compared to a vehicle which was stopping at the junction. With experiment 8, the waiting time at the junction was higher as there was more road traffic which allowed more time to record coordinates. The accuracy of the GPS modules is not perfect and so even when stopped, the module often provides a new location with a few meters difference.

#### 5.2.2. Data Sharing

Whilst the average of recorded coordinates during the experiments was 14, the number that was shared with a base station was much lower, as shown by Table 7.

With every experiment, not all of the coordinates recorded were shared with the base station. The reason for this is simply that once the vehicle had left the range of the base station, it no longer saw any beacons and could not share the data it had. In addition, after leaving the range of the base station it remained within the geofenced area and so it continued to record coordinates. This meant that unless the vehicle was to turn around and re-enter the range of the base station the data could not be shared.

It was expected beforehand that the experiments that saw the vehicle come to a stop would have a higher number of coordinates shared, than those which were moving past at 30 miles per hour. As the data shows, with the exception of experiment 7, there was not much difference between each scenario and the tracking node was able to share all of the data it had recorded up to that point. 

In the case of experiment 7, the traffic generator node being active made communicating successfully whilst moving difficult. This indicates that when there are multiple vehicles present, those which are moving through the junction without stopping will face difficulty in sharing all of their data. Whereas those which are stopped, as experiment 8 shows which also had the traffic generator active, have a greater chance of completing this objective. 

### 5.3. Network Performance

The final task is to evaluate how the network performed during each experiment. With the base station running headless no SSH terminal outputs could be collected and so the data held in Table 8 relies entirely on those collected from the vehicle tracking node.

For the Drive By scenario experiments, an average of 34 beacons was seen by the vehicle tracking node. As the static experiments showed if the tracking node has more than a few coordinates to share, it will have difficulty even if there are no other nodes communicating.

On the other hand, the figure was much higher for the Stop scenario experiments at an average of 58 beacons seen. This figure provides greater opportunity, but that is to be expected as the vehicle is stationary and remains within the range of the base station for longer. 

With all the experiments the resending of packets was required. This increased the number of packets sent overall and added more traffic to the network. This behaviour seemed to mirror what was discovered during the static experimentation, whereupon beacons were missed and in turn acknowledgements. In addition, with experiments 7 and 8, the number of back-off periods greatly increased which again mirrors what was seen when the traffic generator node was used during the static experimentation.

The dynamic experimentation has demonstrated that the artefact system works well in its intended setting, it is capable of collecting coordinate data and sharing it when the opportunity arises. 

The geofence implementation helps to meet one of the project’s objectives, which was to determine a method of collecting data in a manner which was anonymised. The use of sessionIDs and their random generation reduces the chance that a vehicle or individual can be uniquely identified. At the same time though, sessionIDs will still allow for journeys to be stitched together and full routes identified.

The problem though that was exposed during this dynamic experimentation is the fact that not all data is collected. If a vehicle tracking node is not within the range of a base station, then no data can be collected. Even if a tracking node does come within range of one, it may fail to share all of its recorded coordinates. 

There are two factors to discuss in relation to this problem. Firstly, the placement and frequency of the base stations are important. With this experimentation, a single base station was used directly in the centre of the geofence. This meant that the tracking node would only have the opportunity to share coordinates up until that centre point, with any coordinates recorded after going unshared. 

With the geofenced area used in the experimentation, there are only three directions out and so a better strategy could be to use three base stations. One base station on the border of the geofence in each direction. With this configuration, a tracking node entering and exiting the area will always pass by two base stations and more coordinates can be collected. This, of course, depends on the road structure of the area. Future research is needed to understand how placement and frequency of base stations can affect the number of coordinates that are shared versus unshared.

The second aspect of the problem is to do with the storing of coordinates. The coordinates recorded are added to a queue array stored during the lifetime of the Python script in RAM. Once the Raspberry Pi is unpowered the contents of the RAM is lost and so are the coordinates. 

If a vehicle fails to re-enter the range of a base station before the ignition is turned off and the Raspberry Pi loses power, then any unshared data is lost. These experiments were run on the premise that each was a new journey into the geofence and so the script was stopped between each. If this had not been done, then upon seeing a beacon this previous journey’s data could have been shared.

The storage of coordinates was done in this manner to reduce the number of packets that must be shared when a base station comes into range. If the tracking node has to deal with keeping the information about older journeys, then newer data from other nodes have less chance to be shared. Furthermore, if the tracking node experiences limited opportunity to share its data then the queue would grow ever larger. At some point, a line needs to be defined as to whether the data is still relevant.

Whether or not the data is still relevant depends what the artefact system is used for. For instance, if it is used to collect route data with the intention of counting how many vehicles use a particular section of road, such as many of those alternative solutions discussed in Section 2, then the data will always be relevant.

If on the other hand, it is being with an ITS (Intelligent Traffic System) then only the most current data is relevant. Decisions are based on what is currently happening and not what happened a day or so ago. Historical data has some importance but sharing it may take opportunities away from other tracking nodes which have newer, more current data to share.

There is a further point to recognise here. If the system artefact system was used for this purpose, then there is an inherent delay in the information being collected. Once again, a tracking node must find a base station before it can pass its recorded data over, so it can be processed and the according ITS actions applied. This point once again feeds back into the need for further research to understand what effect of placement and frequency of base stations can have.

## 6. Conclusions and Future Work

With the artefact system evaluated and its known advantages and disadvantages, the project was drawn to a close. The project saw the creation of a wireless sensor network that was used to collect GPS location data from vehicles whilst respecting the privacy of its occupants.

Before any of this design and development took place, a systematic literature review of relevant topics was carried out. The research was conducted from two perspectives: the city and the user. The city perspective helped the understanding of how the system should be implemented and what advantages there could be if it was. The user perspective helped to understand how concerned people are with this type of technology and suggested ways of increasing adoption.

A base station was created to be the hub of the wireless sensor network. Utilising a purposely designed media access control protocol, the base station not only collected data from local vehicles but also informed them first that it was present in the form of beacons which acknowledged the data just received. Spaced at two hundred milliseconds, the beacon intervals allowed vehicles enough time to share data whilst maintaining an orderly communication method.

Each vehicle tracking node that shared data with the base station, shared only data gathered from within a geofence. Outside of the software based customisable geofence, no data was collected and, instead, dropped. Each time the vehicle drove into the geofenced area, a new sessionID was randomly generated. This sessionID was used as an identifier for that vehicle and the journey it was currently on. These measures helped to reduce the chance of logs being kept about specific vehicles and their habits or being able to identify individuals. 

A thought towards cost was maintained throughout the project, with both the base station and vehicle tracking node cost kept to a minimum. The difficulty of installing in a vehicle was also kept in mind, with the final implementation of the vehicle tracking node only requiring power from the vehicle to operate.

Whilst this project is complete there is still further work that can be done to improve the solution. This further work has already been alluded to during the evaluation chapter and involves the efficiency of the MAC protocol. Whilst the protocol operates in a satisfactory manner more work is needed to perfect it before it can be used on a larger scale. There also is also a requirement for further work to understand how base station placement and frequency affects how much data can be collected.

## Figures and Tables

**Table 1:** Comparison between the different solutions and this project’s system.

	Intrusive or Non-intrusive Implementation	Gathered Information	Area Covered	Privacy	Lifespan
Pneumatic Road Tube	Intrusive: Requires short-term road closure for the tube to be bolted to surface.	Count	Small: Counts vehicles between junctions.	Full: No identification and record kept of vehicle	Short-term
Video Image Detection	Intrusive: Requires installation of CCTV cameras roadside	Counts, Type of vehicle, Number plates, Partial Route	Small: Can only monitor a small section of the road network	Little: Identification and record kept of vehicle	Long-term
Induction Loop	Intrusive: Requires lane closure for installation	Count, Type of vehicle	Small: Only the immediate area above	Full: No identification and record kept of vehicle	Long-term
Piezoelectric Sensors	Intrusive: Requires lane closure for installation	Count, Type of vehicle	Small: Only the immediate area above	Full: No identification and record kept of vehicle	Long-term
Manual Counting	Non-intrusive: No lane or road closures required	Count, Vehicle type, Number plates	Small: Can only monitor a small section of the road network	Little: Identification and record kept of vehicle	Short-term
Smartphone App	Non-intrusive: No lane or road closures required	Vehicle type, Current location, Full route, Accelerometer	Large: Travels with the user to provide full route data	Little: Identification and movement record kept with identifiers	Long-term
Artefact	Intrusive: Requires installation of base stations roadside	Full route data, Current location	Medium: Travels with the user but provides data only when within the geofence	Medium: Identification and movement record kept with no identifiers	Long-term

**Table 2:** Total Length in Seconds for the First Three Experiments.

Experiment Number	Length (seconds)
1	16.69
2	18.47
3	20.75
Average (s)	18.64

**Table 3:** Total Length in Seconds for the Second Three Experiments.

Experiment Number	Length (seconds)
4	17.17
5	18.77
6	61.13
Average (s)	32.26

**Table 4:** Data from the base station perspective.

Experiment #	Traffic Generator Active	Slots Total	Slots Used	Efficiency
1	No	69	56	81.2%
2	No	81	62	76.5%
3	No	91	59	64.8%
Average Efficiency				74.2%
4	Yes	67	60	89.6%
5	Yes	76	66	86.8%
6	Yes	283	219	77.4%
Average Efficiency				84.6%

**Table 5:** Data from the tracking node perspective.

Experiment #	Traffic Generator Active	Beacons Missed	Resend Totals
1	No	6	9
2	No	14	14
3	No	9	11
4	Yes	3	6
5	Yes	9	9
6	Yes	51	55

**Table 6:** Number of Coordinates Collected during each experiment.

Experiment Number	Scenario	Coordinates Recorded Within Geofence	Coordinates Recorded Outside of Geofence	SessionID
1	Drive By	16	0	3978
2	Stop	13	0	6533
3	Drive By	15	0	5626
4	Stop	13	0	2179
5	Drive By	16	0	1663
6	Stop	13	0	2250
7	Drive By	16	0	5342
8	Stop	15	0	4361

**Table 7:** Percentage of Coordinates Collected Shared with Base Station During Experiment

Experiment Number	Scenario	Coordinates Recorded Within Geofence	Coordinates Shared	Percentage Shared
1	Drive By	16	8	50%
2	Stop	13	7	53.8%
3	Drive By	15	9	60%
4	Stop	13	8	61.5%
5	Drive By	16	10	62.5%
6	Stop	13	8	61.5%
7	Drive By	16	4	25%
8	Stop	15	9	60%
Average Percentage Shared				54.3%

**Table 8:** Network performance during experiments.

Experiment Number	Scenario	Beacons Seen	Coordinates Shared	Packets Sent
1	Drive By	47	8	12
2	Stop	86	7	13
3	Drive By	82	9	14
4	Stop	110	8	13
5	Drive By	87	10	14
6	Stop	126	8	10
7	Drive By	56	4	13
8	Stop	141	9	21

## Appendix A

**Base Station**		**Vehicle Tracking Node**		
**Timestamp**	**Packet Got?** ***1 equals Yes, *** ***0 equals No***	**Timestamp**	**Acknowledgement?** ***1 equals Yes, *** ***0 equals No***	**Back-Off Value**
1538998289285	1	First Message Sent		
1538998289495	1	1538998289495	1	0
1538998289705	1	1538998289705	1	0
1538998289915	1	1538998289915	1	0
1538998290125	1		1	0
1538998290335	1		1	0
1538998290545	1		1	0
1538998290755	1		1	0
1538998290975	1		1	0
1538998291185	0	1538998291185	0	4
1538998291405	0			3
1538998291615	0			2
1538998291825	0			1
1538998292035	1			0
	1		1	0
	1		1	0
	1		1	0
	1		1	0
	1		1	0
	1		1	0
	1		1	0
	1		1	0
	1		1	0
	1		1	0
	1		1	0
1538998294585	0		1	0
1538998294805	1	1538998294805	0	0
	1	1538998295015	1	0
1538998295235	0		1	0
1538998295445	1		0	0
	1	1538998295655	1	0
	1		1	0
	1		1	0
	1		1	0
	1		1	0
1538998296725	0			
1538998296935	1	1538998296935	0	0
1538998297155	0			
	0			
1538998297575	1	1538998297575	0	0
	1	1538998297785	1	0
	1		1	0
	1		1	0
	1		1	0
	1		1	0
	1		1	0
	1		1	0
	1		1	0
	1		1	0
	1		1	0
	1		1	0
	1		1	0
	1	1538998300325	1	0
1538998300545	0			
1538998300755	1	1538998300755	1	0
	1	1538998300966	1	0
	1	1538998301185	1	0
1538998301395	0			
1538998301615	1	1538998301615	0	0
	1	1538998301825	1	0
	1	1538998302035	1	0
1538998302255	0			
1538998302465	1	1538998302465	0	0
1538998302675	0			
1538998302885	1	1538998302885	0	0
	1		1	0
	1	1538998303305	1	0
	1		1	0
	1		1	0
			1	0
*Total Packets:*	*56*	*Total Packets:*	*50*	

**Base Station**		**Vehicle Tracking Node**		
**Timestamp**	**Packet Got?** ***1 equals Yes, *** ***0 equals No***	**Timestamp**	**Acknowledgement?** ***1 equals Yes, *** ***0 equals No***	**Back-Off Value**
1538998349605	1	First Message Sent		
	1	1538998349815	1	0
	1		1	0
1538998350245	0			
	0			
1538998350665	1	1538998350665	0	0
	1	1538998350875	1	0
	1		1	0
	1		1	0
1538998351505	0			
1538998351715	1	1538998351715	0	0
1538998351925	0			
1538998352135	1	1538998352135	0	0
	1	1538998352345	1	0
	1	1538998352565	1	0
1538998352775	0			
1538998352985	1	1538998352985	0	0
1538998353195	0			
1538998353405	1	1538998353405	0	0
	1	1538998353615	1	0
	1	1538998353825	1	0
	1	1538998354035	1	0
1538998354245	0			
1538998354455	1	1538998354455	0	0
	1	1538998354675	1	0
	1	1538998354885	1	0
	1		1	0
	1		1	0
	1		1	0
	1		1	0
	1		1	0
	1		1	0
	1		1	0
	1		1	0
1538998356815	0			
1538998357025	1	1538998357025	0	0
	1	1538998357235	1	0
	1		1	0
	1		1	0
1538998357865	0			
1538998358075	1	1538998358075	0	0
	1	1538998358295	1	0
	1		1	0
	1		1	0
	1		1	0
1538998359155	0		1	0
	0	1538998359365	0	4
	0		0	3
	0		0	2
	0		0	1
1538998360215	1		0	0
	1	1538998360425	1	0
	1		1	0
	1		1	0
	1		1	0
	1		1	0
	1		1	0
1538998361685	0			
1538998361896	1	1538998361896	0	0
	1	1538998362106	1	0
	1		1	0
	1		1	0
1538998362735	0			
1538998362945	1	1538998362945	0	0
1538998363155	0			
1538998363365	1	1538998363365	0	0
	1	1538998363576	1	0
	1		1	0
	1		1	0
	1		1	0
	1		1	0
	1		1	0
	1		1	0
	1		1	0
	1		1	0
	1		1	0
	1	1538998365675	1	0
1538998365886	0			
	0			
1538998366305	1	1538998366305	0	0
	1	1538998366515	1	0
			1	0
*Total Packets:*	*62*	*Total Packets:*	*50*	

**Base Station**		**Vehicle Tracking Node**		
**Timestamp**	**Packet Got?** ***1 equals Yes, *** ***0 equals No***	**Timestamp**	**Acknowledgement?** ***1 equals Yes, *** ***0 equals No***	**Back-Off Value**
1538998633735	1	First Message Sent		
1538998633946	0	1538998633946	0	3
	0		0	2
	0		0	1
	0			
1538998634795	1		0	0
	1	1538998635005	1	0
	1		1	0
	1		1	0
	1		1	0
	1		1	0
	1		1	0
1538998636275	0			
1538998636485	1	1538998636485	0	0
	1	1538998636695	1	0
	1		1	0
	1		1	0
	1		1	0
	1		1	0
	1		1	0
	1		1	0
1538998638175	0			
1538998638385	1	1538998638385	0	0
	1	1538998638595	1	0
	1		1	0
	1		1	0
1538998639235	0			
1538998639445	1	1538998639445	0	0
	1	1538998639655	1	0
	1		1	0
	1		1	0
1538998640295	0			
1538998640505	1	1538998640505	0	0
1538998640716	0		0	3
	0		0	2
	0		0	1
	0		0	0
	0	1538998641566	0	3
	0			
	0		0	2
	0		0	1
1538998642415	1		0	0
	1	1538998642625	1	0
	1		1	0
	1		1	0
	1		1	0
	1		1	0
	1		1	0
	1		1	0
1538998644105	0			
	0	1538998644315	0	0
	0		0	3
	0		0	2
	0		0	1
	0		0	0
	0		0	5
	0		0	4
	0		0	3
	0		0	2
	0		0	1
1538998646445	1		0	0
	1	1538998646656	1	0
	1		1	0
	1		1	0
	1		1	0
1538998647495	0			
1538998647705	1	1538998647705	0	0
	1	1538998647915	1	0
	1		1	0
	1		1	0
	1		1	0
	1		1	0
	1		1	0
	1		1	0
	1		1	0
	1		1	0
	1		1	0
	1		1	0
	1		1	0
	1		1	0
	1		1	0
	1		1	0
1538998651065	0		1	0
	0	1538998651275	0	2
	0		0	1
1538998651695	1		0	0
1538998651905	0			
1538998652115	1	1538998652115	0	0
	1	1538998652325	1	0
	1		1	0
	1		1	0
			1	0
*Total Packets:*	*59*	*Total Packets:*	*50*	

**Base Station**		**Vehicle Tracking Node**		
**Timestamp**	**Packet Got?** ***1 equals Yes, *** ***0 equals No***	**Timestamp**	**Acknowledgement?** ***1 equals Yes, *** ***0 equals No***	**Back-Off Value**
1538999314975	1	First Message Sent		
	1	1538999314975	1	0
	1		1	0
	1		1	0
	1		1	0
	1		1	0
	1		1	0
	1		1	0
	1		1	0
	1		1	0
	1		1	0
	1		1	0
	1		1	0
	1		1	0
	1		1	0
1538999318155	0		1	0
	1	1538999318155	0	2
	1		0	1
	1		0	0
	1	1538999318788	1	0
	1		1	0
	0		1	0
	0		1	0
	0	1538999319645	0	4
	0		0	3
	0		0	2
1538999320485	1			
	1		0	1
	1		0	0
	1		0	0
	1	1538999321335	1	0
	1		1	0
	1		1	0
	1		1	0
	1		1	0
	1		1	0
	1		1	0
	1		1	0
	1		1	0
	1		1	0
	1		1	0
	1		1	0
	1		1	0
	1		1	0
	1		1	0
	1			
	1	1538999324745	0	0
	1	1538999324955	1	0
	1		1	0
	1		1	0
1538999325585	0			
	1	1538999325795	0	0
	1	1538999326005	1	0
	1		1	0
	1		1	0
	1		1	0
	1	1538999326845	0	2
	1		0	1
	1		0	0
	1	1538999327475	1	0
	1		1	0
	1		1	0
	1		1	0
	1		1	0
	1		1	0
	1		1	0
	1		1	0
			1	0
*Total Packets:*	*60*	*Total Packets:*	*50*	

**Base Station**		**Vehicle Tracking Node**		
**Timestamp**	**Packet Got?** ***1 equals Yes, *** ***0 equals No***	**Timestamp**	**Acknowledgement?** ***1 equals Yes, *** ***0 equals No***	**Back-Off Value**
1538999371475	1	First Message Sent		
	1	1538999371475	1	0
	1		1	0
	1		1	0
	1		1	0
	1		1	0
	1		1	0
	1		1	0
	1		1	0
	1		1	0
	1		1	0
1538999373785	0		1	0
1538999373995	1	1538999373995	0	0
	1	1538999374205	1	0
	1		1	0
	1		1	0
	1		1	0
	1		1	0
	1		1	0
	1		1	0
	1		1	0
	1		1	0
	1		1	0
	1		1	0
	1		1	0
	1		1	0
	1		1	0
1538999377145	0			
1538999377355	1	1538999377355	0	0
	1	1538999377565	1	0
	1		1	0
	1		1	0
1538999378205	0			
1538999378415	1	1538999378415	0	0
	1	1538999378625	1	0
	1		1	0
	1		1	0
	1		1	0
	1		1	0
	1		1	0
	1			
	1	1538999380105	0	0
	1	1538999380315	1	1
	1		1	1
	1		1	1
	1		1	1
	1		1	1
	1		1	1
	1		1	1
	1		1	1
	1		1	1
	1		1	1
	1			
	1	1538999382655	0	0
1538999382866	0		0	4
1538999383085	1		0	3
	1			
1538999383505	0		0	2
	0		0	1
1538999383925	1		0	0
	1	1538999384145	1	0
	1		1	0
1538999384565	0			
1538999384775	1	1538999384775	0	0
	1		0	4
1538999385195	0			
1538999385405	1			
	1		0	3
1538999385825	0		0	2
1538999386035	1		0	1
	1	1538999386245	0	0
	1	1538999386455	1	0
1538999386665	0			
1538999386875	1	1538999386875	0	0
	1	1538999387085	1	0
	1		1	0
			1	0
*Total Packets:*	*66*	*Total Packets:*	*50*	

**Base Station**		**Vehicle Tracking Node**		
**Timestamp**	**Packet Got?** ***1 equals Yes, *** ***0 equals No***	**Timestamp**	**Acknowledgement?** ***1 equals Yes, *** ***0 equals No***	**Back-Off Value**
1538999448065	1	First Message Sent		
1538999448275	0	1538999448275	0	4
1538999448485	1			
	1		0	3
	1		0	2
	1		0	1
	1			
	1		0	0
	0			
	1		0	0
	1	1538999450175	1	0
	1		1	0
	0		1	0
	0	1538999450805	0	3
	0		0	2
1538999451225	1			
	1		0	1
	1			
	1		0	0
	1	1538999452065	1	0
	1		1	0
	1		1	0
	1	1538999452695	0	4
	0		0	3
	0		0	2
	0		0	1
1538999453535	1		0	0
	1	1538999453745	1	0
1538999453955	0			
1538999454165	1			
	1	1538999454375	0	0
	1		0	3
	1		0	2
	1		0	1
	1		0	0
	1		0	5
	1		0	4
	0		0	3
	0		0	2
	1		0	1
	1		0	0
	0	1538999457125	1	0
	1		1	0
	1			
	1	1538999457765	0	0
	0	1538999457975	1	0
	1		1	0
	1	1538999458395	0	0
	1		0	5
	1		0	4
	1		0	3
	0		0	2
	1		0	1
	1		0	0
	1			
	1		0	0
	1			
	1			
	1		0	0
	1		0	4
	1		0	3
	1		0	2
	1			
	1		0	1
	1		0	0
	1		0	5
	0		0	4
	1		0	3
	1		0	2
	1		0	1
	1		0	0
	1	1538999463235	1	0
	1		1	0
1538999463655	0			
	1	1538999463865	0	0
	1	1538999464085	1	0
	1			
	1	1538999464715	0	0
	0		0	0
	0		0	4
	0		0	3
	1			
	1		0	2
	1		0	1
	1		0	0
	1		0	5
	0		0	4
	0		0	3
	1		0	2
	0		0	1
1538999467255	1		0	0
	1	1538999467465	1	0
	1		1	0
	1		1	0
	1		1	0
	1		1	0
	1	1538999468525	0	3
	1		0	2
	1		0	1
	1		0	0
	1		0	3
1538999469575	0		0	2
	1		0	1
	1		0	0
	1	1538999470216	1	0
	1		1	0
	0			
	1	1538999470855	0	0
	1		0	2
	0		0	1
	1		0	0
	1	1538999471705	1	0
	1		1	0
	1		1	0
	1		1	0
	1		1	0
	0			
	1	1538999472985	0	0
	0			
	1		0	0
	1			
	1		0	0
1538999474035	0		0	5
	1		0	4
	1		0	3
	1		0	2
	0		0	1
	1		0	0
	1		0	3
	1		0	2
	1		0	1
	1		0	0
	1		0	2
	1			
	1		0	1
	1		0	0
	1		0	3
	0		0	2
	0		0	1
	1		0	0
	1	1538999477835	1	0
	1		1	0
	1			
	1	1538999478465	0	0
	1			
	1		0	0
	0		0	0
	1			
	1		0	3
	1		0	2
	1		0	1
	1		0	0
	0			
1538999480566	1			
	1		0	2
	1		0	1
	1			
	0			
	1		0	0
	1	1538999481825	1	1
	1	1538999482035	0	3
	1		0	2
	0		0	1
	1		0	0
	0			
	1	1538999483295	1	0
	1		1	0
	1		1	0
	1			
	1	1538999484135	0	0
	1		0	0
	1			
	1			
	1		0	5
	1		0	4
	0		0	3
	1		0	2
	1		0	1
	1		0	0
	1		0	5
	0		0	4
	0		0	3
	0		0	2
	0		0	1
	1			
	1		0	0
1538999487495	1	1538999487495	1	0
	1		1	0
	0			
	1	1538999488125	0	0
	1		0	3
	0			
	1		0	2
	1			
	1		0	1
	1			
	1		0	0
	1		0	5
	1		0	4
	1		0	3
	1		0	2
	1		0	1
	0		0	0
	1		0	2
	1		0	1
	1		0	0
	0	1538999491275	1	0
	1		1	0
	1	1538999491905	0	0
	1	1538999492115	1	0
	1		1	0
	1	1538999492535	0	5
	1		0	4
	1		0	3
	1		0	2
	1		0	1
	1		0	0
	1		0	5
	1		0	4
	1		0	3
	1		0	2
	1		0	1
	1			
	1			
	1			
	1		0	0
1538999495895	0		0	0
	1			
	1			
	1			
	1			
	1		0	4
	0		0	3
	0			
	1			
	0		0	2
	1		0	1
	1		0	0
	1		0	3
	1		0	2
	1		0	1
	0		0	0
	0		0	5
	0		0	4
	0		0	3
	0		0	2
	1		0	1
	0		0	0
	1			
	0	1538999500305	1	0
	0	1538999500515	0	0
	0	1538999500725	1	0
	1			
	1			
	1			
	1		1	0
	1		1	0
	0		1	0
	1	1538999502195	0	0
	1		0	4
	0			
	1		0	3
	0		0	2
	1		0	1
	1		0	0
	1		0	3
	1			
	1		0	2
	1		0	1
	1		0	0
	1		0	5
	0		0	4
	1		0	3
	1		0	2
	1		0	1
	1		0	0
	1			
	1	1538999506186	1	0
	1		1	0
	0		1	0
	0		1	0
	1		1	0
	1		1	0
			1	0
*Total Packets:*	*219*	*Total Packets:*	*50*	

## Appendix B

**Timestamp**	**Within Fence?**	**Shared?**	**SessionID**	**Recorded Coordinates**	**Send Attempts until Success**
14:15:41	No	No			
14:15:46	No	No			
14:15:51	Yes	Yes	3978	52.06651439, −0.30822246	2
14:15:56	Yes	Yes	3978	52.06626717, −0.30927877	2
14:16:01	Yes	Yes	3978	52.06605841, −0.31038304	1
14:16:06	Yes	Yes	3978	52.06584746, −0.3114968	1
14:16:11	Yes	Yes	3978	52.06564751, −0.31256447	2
14:16:16	Yes	Yes	3978	52.06547095, −0.31360452	1
14:16:21	Yes	Yes	3978	52.06537098, −0.31455171	1
14:16:26	Yes	Yes	3978	52.0653315, −0.31541419	2
14:16:31	Yes	No			
14:16:36	Yes	No			
14:16:41	Yes	No			
14:16:46	Yes	No			
14:16:51	Yes	No			
14:16:56	Yes	No			
14:17:01	Yes	No			
14:17:06	Yes	No			
14:17:11	No	No			
14:17:16	No	No			

**Timestamp**	**Within Fence?**	**Shared?**	**SessionID**	**Recorded Coordinates**	**Send Attempts until Success**
14:20:17	No	No			
14:20:22	No	No			
14:20:27	Yes	Yes	6533	52.06308585, −0.31412471	1
14:20:32	Yes	Yes	6533	52.06368297, −0.31448173	1
14:20:37	Yes	Yes	6533	52.06426095, −0.31478351	1
14:20:42	Yes	Yes	6533	52.06480673, −0.31501752	1
14:20:47	Yes	Yes	6533	52.06520865, −0.31517696	2
14:20:52	Yes	Yes	6533	52.06532235, −0.31524813	3
14:20:57	Yes	Yes	6533	52.06531337, −0.3149738	2
14:21:02	Yes	Yes	6533	52.06539673, −0.31436448	2
14:21:07	Yes	No			
14:21:12	Yes	No			
14:21:17	Yes	No			
14:21:22	Yes	No			
14:21:27	Yes	No			
14:21:32	No	No			
14:21:37	No	No			

**Timestamp**	**Within Fence?**	**Shared?**	**SessionID**	**Recorded Coordinates**	**Send Attempts until Success**
14:27:17	No	No			
14:27:22	No	No			
14:27:27	Yes	Yes	5626	52.06653354, −0.30817213	1
14:27:32	Yes	Yes	5626	52.06625955, −0.30933655	1
14:27:37	Yes	Yes	5626	52.06600928, −0.31058485	2
14:27:42	Yes	Yes	5626	52.0657402, −0.31186975	2
14:27:47	Yes	Yes	5626	52.06552941, −0.31310313	1
14:27:52	Yes	Yes	5626	52.06538453, −0.31416301	2
14:27:57	Yes	Yes	5626	52.06532625, −0.31502989	1
14:28:02	Yes	Yes	5626	52.06526948, −0.3157568	2
14:28:07	Yes	Yes	5626	52.06518256, −0.31645119	2
14:28:12	Yes	No			
14:28:17	Yes	No			
14:28:22	Yes	No			
14:28:28	Yes	No			
14:28:33	Yes	No			
14:28:38	Yes	No			
14:28:43	No	No			
14:28:48	No	No			

**Timestamp**	**Within Fence?**	**Shared?**	**SessionID**	**Recorded Coordinates**	**Send Attempts until Success**
14:31:35	No	No			
14:31:40	No	No			
14:31:45	Yes	Yes	2179	52.06334934, −0.31431263	1
14:31:50	Yes	Yes	2179	52.0639734, −0.31466067	1
14:31:55	Yes	Yes	2179	52.06458848, −0.31493415	2
14:32:00	Yes	Yes	2179	52.06509055, −0.31515036	2
14:32:05	Yes	Yes	2179	52.06526948, −0.31524525	1
14:32:10	Yes	Yes	2179	52.0653032, −0.31523644	1
14:32:15	Yes	Yes	2179	52.0653581, −0.31491941	2
14:32:20	Yes	Yes	2179	52.06543028, −0.31425366	3
14:32:25	Yes	No			
14:32:30	Yes	No			
14:32:35	Yes	No			
14:32:40	Yes	No			
14:32:45	Yes	No			
14:32:50	No	No			
14:32:55	No	No			

**Timestamp**	**Within Fence?**	**Shared?**	**SessionID**	**Recorded Coordinates**	**Send Attempts until Success**
14:38:06	No	No			
14:38:11	No	No			
14:38:16	Yes	Yes	1663	52.0665637, −0.30806064	2
14:38:21	Yes	Yes	1663	52.0662831, −0.30921286	1
14:38:26	Yes	Yes	1663	52.06605503, −0.31042083	1
14:38:31	Yes	Yes	1663	52.06581441, −0.31171183	1
14:38:36	Yes	Yes	1663	52.0655938, −0.31292674	2
14:38:41	Yes	Yes	1663	52.06543062, −0.31405914	1
14:38:46	Yes	Yes	1663	52.06536725, −0.31508309	2
14:38:51	Yes	Yes	1663	52.06528931, −0.31599589	2
14:38:56	Yes	Yes	1663	52.0652239, −0.31674551	1
14:39:01	Yes	Yes	1663	52.06519984, −0.31681798	1
14:39:06	Yes	No			
14:39:11	Yes	No			
14:39:16	Yes	No			
14:39:22	Yes	No			
14:39:27	Yes	No			
14:39:32	Yes	No			
14:39:37	No	No			
14:39:42	No	No			

**Timestamp**	**Within Fence?**	**Shared?**	**SessionID**	**Recorded Coordinates**	**Send Attempts until Success**
14:42:27	No	No			
14:42:32	No	No			
14:42:37	Yes	Yes	2250	52.06343491, −0.31435431	1
14:42:42	Yes	Yes	2250	52.06407354, −0.31469642	2
14:42:47	Yes	Yes	2250	52.06468863, −0.31497041	1
14:42:52	Yes	Yes	2250	52.06516188, −0.31517391	1
14:42:57	Yes	Yes	2250	52.06528304, −0.3152278	1
14:43:03	Yes	Yes	2250	52.06531286, −0.31521306	1
14:43:08	Yes	Yes	2250	52.06536234, −0.31484435	2
14:43:13	Yes	Yes	2250	52.06544977, −0.31411302	1
14:43:18	Yes	No			
14:43:23	Yes	No			
14:43:28	Yes	No			
14:43:33	Yes	No			
14:43:38	Yes	No			
14:43:43	No	No			
14:43:48	No	No			

**Timestamp**	**Within Fence?**	**Shared?**	**SessionID**	**Recorded Coordinates**	**Send Attempts until Success**
14:49:01	No	No			
14:49:06	No	No			
14:49:11	Yes	Yes	5342	52.06647203, −0.30838783	2
14:49:16	Yes	Yes	5342	52.06624277, −0.30953311	9
14:49:21	Yes	Yes	5342	52.06602978, −0.31077514	1
14:49:27	Yes	Yes	5342	52.06573257, −0.31235622	1
14:49:32	Yes	No			
14:49:37	Yes	No			
14:49:42	Yes	No			
14:49:47	Yes	No			
14:49:52	Yes	No			
14:49:57	Yes	No			
14:50:02	Yes	No			
14:50:07	Yes	No			
14:50:12	Yes	No			
14:50:17	Yes	No			
14:50:22	Yes	No			
14:50:27	Yes	No			
14:50:32	No	No			
14:50:37	No	No			

**Timestamp**	**Within Fence?**	**Shared?**	**SessionID**	**Recorded Coordinates**	**Send Attempts until Success**
14:53:24	No	No			
14:53:29	No	No			
14:53:34	Yes	Yes	4361	52.06317498, −0.31419232	1
14:53:39	Yes	Yes	4361	52.06377685, −0.31452935	2
14:53:44	Yes	Yes	4361	52.06433805, −0.31479809	1
14:53:49	Yes	Yes	4361	52.06485536, −0.31503938	1
14:53:54	Yes	Yes	4361	52.06516866, −0.31516595	2
14:53:59	Yes	Yes	4361	52.06523661, −0.31522543	11
14:54:04	Yes	Yes	4361	52.06523949, −0.31522373	1
14:54:09	Yes	Yes	4361	52.06530015, −0.31508123	1
14:54:14	Yes	Yes	4361	52.06534387, −0.31455781	1
14:54:19	Yes	No			
14:54:24	Yes	No			
14:54:29	Yes	No			
14:54:34	Yes	No			
14:54:39	Yes	No			
14:54:44	Yes	No			
14:54:49	No	No			
14:54:54	No	No			
